# A mobile intervention to reduce pain and improve health-III: protocol for a remotely delivered randomized controlled trial of physical activity for pain management in older adults with obesity and knee or hip osteoarthritis

**DOI:** 10.3389/fdgth.2026.1739501

**Published:** 2026-05-21

**Authors:** Jason Fanning, Deja O. Dobson, Sherri A. Ford, Megan Bennett, Iris Leng, Anna C. Martin, James Merritt, Fancis J. Keefe, W. Jack Rejeski, Amber K. Brooks

**Affiliations:** 1 Department of Health and Exercise Science, Wake Forest University, Winston Salem, NC, United States; 2 Biostatistics and Data Science, Wake Forest University School of Medicine, Winston Salem, NC, United States; 3 Department of Psychiatry and Behavioral Science, Duke University, Durham, NC, United States; 4 Department of Anesthesiology, Wake Forest University School of Medicine, Winston Salem, NC, United States

**Keywords:** behavior change, older adult, physical activity, sedentary behavior, technology

## Abstract

As many as three in four older adults live with chronic pain, and osteoarthritis is a leading cause of chronic pain in older adults. Knee and hip osteoarthritis being the most common forms of the condition. Osteoarthritis symptoms are worsened by low levels of physical activity, excess sustained sedentary time, weight gain, and social isolation, ultimately impairing quality of life. Data from several pilot trials have demonstrated the feasibility, acceptability, and potential benefit of a unique remote group-mediated behavioral intervention rooted in social cognitive and self-determination theories that targets three domains of behavior change: (1) dietary behavior change with a focus on weight loss via caloric restriction alongside diet quality, satiety, and reduced inflammation, (2) increased physical activity, and (3) decreased sitting via the accumulation of steps in frequent bouts throughout the day (i.e., daylong movement). Herein we describe the protocol for a Stage-II parallel randomized controlled trial examining the efficacy of 6 months of a remotely delivered group-mediated daylong movement and weight loss intervention in older adults with obesity and chronic knee or hip osteoarthritic pain. Outcomes of interest include daily steps (primary outcome) and pain interference, body weight, and physical function (secondary outcomes). We will also explore intervention effects on long-term behavior change over 12 months following the intervention and whether changes in steps, body weight, pain catastrophizing, or pain self-efficacy mediate intervention effects on pain interference, if present. Low-active older adults (*N* = 200) with chronic osteoarthritic hip and/or knee pain and obesity will be randomly assigned to the daylong movement and weight loss intervention or to an enhanced usual care control. All participants will receive the same self-monitoring technologies to account for any effect of basic device provision on activity and diet behavior. The results of this trial will inform future real-world efficacy and effectiveness trials of a package well-suited to broad scale delivery.

## 1 Introduction

Osteoarthritis (OA) is a leading cause of chronic pain in older adults that promotes physical inactivity and declining quality of life. The prevalence of OA has more than doubled since 1990, with hip and knee OA representing the most common forms of the condition (1, 2). Chronic pain is also costly: in a 2012 report, Gaskin and Richard (3) found the incremental medical costs associated with chronic pain in adults and older adults totaled between $261–$300 billion annually.

**Figure 1:**
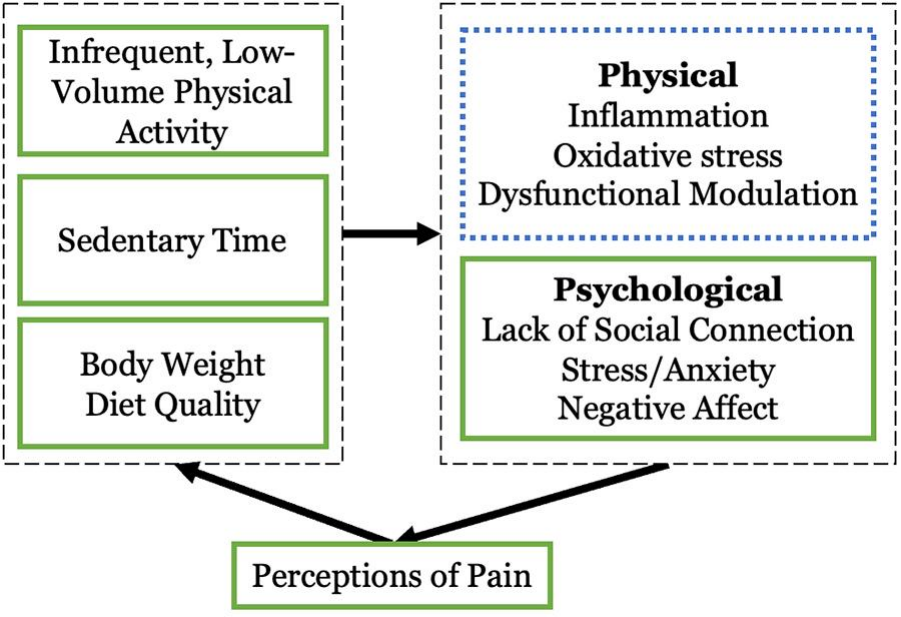
The cycle of movement dysregulation, excess body weight, and perceptions of pain. **Solid green** boxes are addressed in the proposed project, **blue dotted** are candidates for future research.

Accumulating evidence suggests that addressing the interrelated behavioral targets of physical activity (PA) promotion, sustained sitting time reduction, and dietary weight management may help to improve pain symptoms in older adults with chronic pain (4–7). Figure 1 demonstrates a simplified cyclical pathway between these lifestyle factors and perceptions of pain. Excess body weight, an inflammatory diet, and the presence of prolonged periods of sitting paired with infrequent and/or low levels of light intensity PA (LPA) and moderate-to-vigorous intensity PA (MVPA) are consistently associated with the experience of pain. Knee and hip OA pain appear especially responsive to PA, sedentary behavior, and body weight (8, 9), with emerging evidence suggesting benefit of an anti-inflammatory diet for OA pain (10, 11). The American College of Rheumatology/Arthritis Foundation Guidelines for the management of OA (12) strongly recommend increasing PA—especially in combination with dietary weight loss (WL)—for knee and hip OA. They also note there is insufficient evidence as to the optimal PA prescription for these conditions, as most evidence suggests that most types of PA, alongside reduced sedentary time, are associated with better outcomes in OA. For instance, White and colleagues demonstrated that more daily steps are associated with lower risk of functional limitations for those with knee OA, and that 6,000 steps/day discriminated those who did and did not develop functional limitations over a 2-year period (13, 14). Those living with knee OA spend approximately 2/3 of their waking time sedentary, and this is associated with poorer physical functioning even after accounting for daily MVPA time (8). In another study of participants with chronic knee pain arising primarily from OA in the Korean National Health and Nutrition Examination Survey cohort, Lee and colleagues (15) found that more sedentary behavior was associated with worse knee pain, and while higher levels of PA were associated with less knee pain, those with excess sitting time (>10 h/day) and high levels of PA were more likely to experience chronic knee pain. Likewise, those with obesity were more likely to have knee pain. The authors concluded that targeting PA, sedentary behavior, and obesity is crucial for managing chronic knee pain.

A strong body of evidence supports that structured exercise paired with dietary weight loss improves a host of clinically relevant outcomes in OA, including physical functioning (16–18). However, data from adult and older adult populations suggests that a structured exercise approach to promoting PA can contribute to compensatory increases in sedentary behavior and decreases in non-exercise PA in the short term, and poor rates of long-term maintenance of behavior change (19–21). The current project builds upon our National Institutes of Health (NIH) Stage I pilot work [a Mobile Intervention to Reduce Pain and Improve Health (MORPH) and MORPH-II] (5, 7, 22, 23). In these studies, we developed and refined a unique behavioral intervention prioritizing (1) achieving a greater volume of daily PA by (2) moving often throughout the day in enjoyable activities (i.e., “daylong movement”), thus indirectly breaking up sustained sitting and avoiding pain due to overexertion, paired with (3) healthy eating with a caloric deficit sufficient to elicit 8%–10% reduction in body mass over the study period. We would briefly emphasize that the emphasis on daylong movement is primarily a behavioral approach designed to effect change one's overall daily volume of activity. Focusing on daylong movement teaches participants that they can achieve their activity goals through enjoyable and practical lifestyle activities. Additionally, interrupting sustained sitting and avoiding overexertion can help to avoid behavioral lapses associated with acute pain, which is naturally an important barrier to further activity in those living with chronic pain. In this way, the intervention is in alignment with the “move more, more often” recommendation underlying the US physical activity guidelines and our own prior research (24–26), which underscore that the most robust health effects are accrued by first simply increasing one's volume of activity in bouts of any duration; and then secondarily encouraging early and frequent movement.

It should be emphasized that at the present time, there are few robust protocols targeting the promotion of physical activity across the day; none leverage evidence-based digital health tools to provide remote intervention, ongoing social connection, and real-time feedback on activity patterns and weight loss; and none target chronic pain except for those from our team. For instance, Jones and colleagues recently conducted a systematic review of trials testing brief bouts of activity distributed across the day. They identified 32 studies and found some initial evidence for benefit to cardiovascular fitness but emphasized that this evidence arose from a limited number of small quasi-experimental studies, small randomized controlled trials, or qualitative studies. None of the included studies targeted pain outcomes (27). Relatedly, there are no large randomized trials deploying a sedentary behavior intervention specifically for older adults with chronic pain. It is also important to recognize that daylong activity promotion meaningfully differs from many sedentary behavior reduction interventions, which place emphasis—via educational content, intervention strategy, and behavioral feedback—on avoiding sitting, often via standing, rather than on strategies for building an active lifestyle (e.g., encouraging the development and maintenance of a repertoire of enjoyable or practical daily activities) (28). What follows is a description of the *Mobile Intervention to Reduce Pain and Improve Health-III (MORPH-III)* trial (ClinicalTrials.org: NCT06623669). MORPH-III aims to test the efficacy of the remotely delivered MORPH intervention for improving daily steps as an index of overall daily activity volume (primary outcome), reducing pain interference and body weight, and improving physical function (secondary outcomes) over 6 months among older adults with obesity and chronic pain due to knee or hip OA. We will also explore for relationships between patterns of daily activity (e.g., via conventional measures such as daily sedentary breaks as well as via functional data analysis methods) and changes in secondary outcomes (7, 26). Based upon our prior work (5, 7, 29), we hypothesized those who received the MORPH intervention will (1) demonstrate a significant increase in steps relative to control after 6 months; and (2) report significant reductions in pain interference and body weight and improvements in physical function relative to control after 6 months.

## 2 Methods

### 2.1 Study overview

MORPH-III is designed as an NIH Stage-II assessor-blind parallel randomized controlled trial. A total of 200 older adults (aged 65 and older) with chronic pain and obesity will be randomly assigned in a 1:1 ratio, stratified by sex, to either a 6-month remote group behavioral intervention or the control group receiving enhanced usual care. The randomization table will be generated by an unblinded statistician in SAS (version 9.4), with randomization occurring in REDCap by an unblinded staff member once the participant is consented and completes baseline testing procedures. To examine the durability of behavior change, participants will be followed across a 12-month maintenance period, for a total study duration of 18 months, during which they will retain access to all technological tools.

### 2.2 Selection and enrollment of participants

Recruitment efforts will target low-active older adults (aged 65 and older) with obesity and chronic knee/hip OA pain but no contraindications for unsupervised PA as determined via the *Exercise Assessment Screening for You* (EASY) tool (30, 31) (see the Supplementary Material for a full list of inclusion and exclusion criteria). Low-active individuals are those not engaging in any weekly resistance training and/or 20 or more minutes of aerobic exercise on 2 or more days per week in the previous 12 weeks. This will be determined using aerobic and resistance exercise items from the Community Healthy Activities Model Program for Seniors (CHAMPS) questionnaire (32) modified to reflect the previous 12 weeks. Obesity status [i.e., body mass index (BMI) ≥ 30 kg/m^2^] will be ascertained via self-report during phone screening and corrected via the Shields equation (33). OA status will be assessed by phone using the validated Roux questionnaire based upon the American College of Rheumatology knee or hip OA criteria (34). Those reporting shortness of breath, chest pain, dizziness with walking, standing up, or stair climbing, or with uncontrolled hypertension or at high fall risk must receive approval from their primary care physician to participate in the study. The modified telephone interview for cognitive status (TICS-M) (35) will also be administered during the phone screening. This cognitive screener taxes various aspects of cognitive functioning and produces a score out of 50 such that higher scores indicate better cognitive functioning. Scores of 32 or better are considered cognitively normal and as such, scores less than 32 are exclusionary in this study (35). Eligible individuals will not be taking weight loss medications at time of enrollment. A community advisory board (CAB) will be empaneled to achieve a generalizable sample by closely matching population distributions reflected within census data for our recruitment areas. We will recruit participants using community outreach, targeted mailings, newsletter and mailing list resources maintained by our medical center, targeted digital advertisements, and medical record recruitment methods such as medical record screening and patient portal messaging. Finally, we hope to identify additional recruitment outlets via collaboration with the CAB.

Those who respond to recruitment strategies will be provided with a study description and short screening via phone with a study staff member to determine eligibility. Those eligible will engage in a baseline virtual testing appointment following procedures piloted in MORPH-II (6, 7). Specifically, participants without a webcam-enabled device will receive a tablet computer via mail (cellular enabled if required) and will complete brief phone-based self-efficacy-driven technology orientation. This training is led by a study staff member, typically lasting between 30 and 60 min. This testing kit will also contain an easy-to-use point-and-shoot camera with tripod, a rope to mark a 4 m walking course, and instructions for positioning and operating the camera and for completing the physical function tasks. Before any data collection, participants will provide digital informed consent to participate in line with the 1964 Declaration of Helsinki. Those who qualify for the study and provide consent will complete the remainder of baseline testing, including functional testing, via video conference, using an institutional instance of Zoom, under the supervision of testing staff. All study procedures are approved by an institutional review board (Wake Forest University Institutional Review Board, IRB00128035) and are pre-registered at clinicaltrials.gov (NCT06623669).

### 2.3 Study interventions

All participants will receive a group-specific intervention technology kit (5, 7, 29) to facilitate a consistent participant experience, regardless of whether they have access to an internet-enabled smart device. For all participants this kit includes study self-monitoring technologies (i.e., a Fitbit Charge activity monitor and BodyTrace wireless weight scale). Those randomized to the intervention condition will also receive an iPad tablet computer (cellular data enabled if required) equipped with all study-required applications to facilitate participation in the intervention.

#### 2.3.1 Enhanced usual care control

As noted above, those assigned to the control condition will receive study self-monitoring technologies (the Fitbit activity monitor and BodyTrace wireless weight scale) to account for any short-term impact on activity and weight management behaviors associated with receipt of self-monitoring technologies. Upon completion of the 18-month study period, control participants will be offered a print and digital version of intervention workbook materials, and they will be allowed to keep the Fitbit monitor. At each testing timepoint (baseline, 6 months, 18 months), control participants who do not have access to a webcam-enabled home computer will also receive an iPad to complete assessments and will then return the tablet to the study team via prepaid post.

#### 2.3.2 The MORPH intervention

The MORPH daylong PA and dietary weight loss intervention is fully remotely delivered and includes a blend of weekly group meetings and one-on-one coaching sessions (see Table 1 for key components). The intervention in supported by a set of digital health technologies designed to provide real time feedback on progress toward activity and weight goals, cue participants to notice successes as they occur, and allow for connection between participants and group leaders between sessions. What follows is a detailed description of weekly group meetings, individual coaching sessions, the activity and dietary goals, and digital health technologies supporting the MORPH intervention.

**Table 1:** Key MORPH intervention components at a glance.

Component	Description
Weekly Group Sessions	Delivered via videoconference
	Based in social cognitive and self-determination theories and principles of group dynamics.
	Allows for development of social connection, sharing successes, troubleshooting challenges, learning and practicing key skills required for health behavior change
Individual Coaching Sessions	Delivered via phone or videoconference
	Troubleshooting behavioral or technical issues
	Goal review and revision
Physical Activity Goal Setting	Daily goals include both objective (cumulative steps) and qualitative (distribution of steps) targets
	Based upon ActivPAL and Fitbit stepping data
	Aim to progress by approximately 20% weekly to achieve at least 7,000 daily steps
Body Weight Goals	Reduction of ∼6% over 6 months; ∼10% over 18 months
	Based upon data from the BodyTrace scale
	Focus on eating for vitality and reduction of inflammation
Companion App	An iPad app designed to provide real-time feedback on activity and weight goals, cue awareness of successes as they occur, and facilitate social connection between meetings.

##### 2.3.2.1 Weekly group meetings

Social connection is central to contemporary theories of behavior change (36, 37), and is vital to the success of behavior change programming. In well-designed group-mediated programs, the group often becomes a potent source of self-efficacy, offering verbal encouragement and vicarious experience of success, and acting as a source of barrier-reduction strategies (38). These sessions last approximately one hour and are delivered to groups of approximately 8–12 participants to allow for easy group discussion and to buffer against very small group sizes in the event of absence or illness. Participants complete the study in cohorts, following a series of predefined sessions guided by the MORPH workbook. All MORPH sessions will be led by an experienced behavioral interventionist trained by the study principal investigator, with some nutrition content covered by an institutional registered dietician. Most group meetings will include a very brief social bonding activity wherein small groups of 2–3 participants will be split into breakout rooms to talk through a series of PA, diet, and pain-related prompts designed to foster increasing self-disclosure following recommendations by Aron and colleagues (39). After 5–10 min, participants will return to the group meeting to discuss didactic content from a study workbook, share progress toward individual goals, troubleshoot challenges as they arise, and finish with mindfulness activities modeled on mindfulness-based relapse prevention (40). Didactic content will include a focus on how achieving sufficient PA while avoiding sustained sitting relates to pain, and how reducing body weight can improve pain symptoms. Participants will learn and practice self-regulatory skills required for successful behavior change during weekly meetings and will learn to manage the influence of pain and other psychosocial states (e.g., stress and negative affect) on their health behaviors. During the 12-month maintenance period, participants will retain access to the workbook and study technologies and will be encouraged to sustain their PA and dietary behavior change.

##### 2.3.2.2 Individual coaching calls

In addition to weekly group meetings, participants in the active intervention will also receive brief (i.e., approximately 15 min, though this varies depending on need) weekly one-on-one coaching calls. The primary purpose of these calls is to cue participants to deeply inspect their daily feedback provided within the *Companion* App (described below). This approach was added following earlier daylong PA studies in response to the finding that participants found it difficult to re-conceptualize PA as something other than a single bout of challenging exercise. Additionally, it is well-documented that individuals underestimate time spent sedentary (41), and this lack of awareness makes modifying extended sedentary bouts difficult. During individual coaching calls, the interventionist guides the participant through their graphical daily daylong PA and weight loss feedback, reinforcing areas of success (e.g., weights within range or periods with frequent movement) and generating strategies for addressing unsuccessful periods. During each call, the interventionist will complete a checklist capturing all key elements of the call, including review of their *Companion* App data, review of previous week's goals (including number of days on which goals were achieved), and what the participant and interventionist decided upon for the next week's goals. Note that these calls may be led by the dietitian if the participant has nutrition-specific concerns.

##### 2.3.2.3 Daylong physical activity goals

Intervention participants will work to achieve the dual goals of increasing daily steps while accumulating these steps throughout the day via enjoyable or satisfying activities. Focusing on *the accumulation* of PA encourages participants to break sustained bouts of sitting. Weekly sessions help participants to learn the value of achieving a sufficient volume of PA—especially activities that are of a moderate intensity such as active transport or structured exercise as tolerated—with an emphasis on developing a repertoire of intrinsically enjoyable activities to account for day-to-day changes in preferences and access to active resources. The group allows participants to collaboratively develop enjoyable PA repertoires, demonstrate competency, view the successes of others, and revise maladaptive expectancies (e.g., that PA must be aversive to be effective). Participants also engage with visual feedback in the *Companion App* several times each day to better understand their progress toward daily stepping goals and how their activity is patterned across the day.

##### 2.3.2.4 The selection of steps as the physical activity target

PA recommendations suggest older adults engage in the equivalent of at least 150 min of moderate-intensity PA each week in bouts of any duration (25, 42). Recognizing the value of steps as an easily understood and measured metric, Tudor-Locke and colleagues proposed a step translation of this goal. They suggest a prescription of 7,000–10,000 steps per day approximates the addition of 30 min of daily MVPA to habitual activities of daily living among older adults (43); a target that exceeds step recommendations for sustaining physical function in OA (13, 44). Recent evidence from large national studies indicates mortality is associated with step count but not intensity (45, 46). Step goals are also well-suited to daylong PA interventions, allowing individuals to monitor patterns of stepping and inactivity via available technologies. Walking is also the most preferred mode of PA among older adults (47) and provides an excellent platform for exploring a variety of enjoyable activities (e.g., walking for exercise or transport, hiking), many of which can be social in nature. In MORPH-III, participants will aim to achieve a long-range daylong PA goal of at least 7,000 daily steps on 6 days per week by the end of the initial 6-month period. Participants will aim to sustain program-related improvements in stepping during the maintenance period. Step goals will be set initially based upon baseline PA data, with an initial weekly goal of ∼20% increase over baseline ActivPAL-recorded steps (e.g., adding 800 daily steps for those achieving 4,000 steps per day). Subsequent goals will be refined in collaboration with the interventionist with the aim of increasing by approximately 20% weekly. This percentage will taper as individuals achieve greater daily steps and ultimately transition to maintenance goals as individuals stabilize above 7,000 daily steps. At this point, participants will continue to focus on distributing their steps throughout the day and on continuing to grow their activity repertoire.

##### 2.3.2.5 Dietary weight loss goals

Intervention participants will receive a behavioral intervention focused on reducing caloric intake sufficient to produce a target WL of ∼6% of initial body mass in 6 months, and ∼10% over the full 18-month period. Generally, goals will be prescribed to achieve an energy deficit of approximately 400 kcal/d from daily weight maintenance energy requirements following procedures used in prior trials (48, 49). Calorie goals will not be set below 1,100 kcal/day for women or 1,200 kcal/day for men. Macronutrient goals will include a targeted intake of 20% of calories from protein, 25% from fat, and 55% from carbohydrates. Weekly group meetings will include both didactic and experiential content related to eating healthfully within a reduced calorie diet, better understanding and estimating portion sizes, the impact of momentary psychological and social states on food choices, and the acquisition of self-monitoring skills, including the use of the food logging to monitor dietary intake, and the use of the Companion App to monitor trajectories of weight loss over time. Participants will be asked to weigh daily using their BodyTrace wireless scale throughout the 6-month intervention period, and to utilize print or digital food logging for at least the first month of the intervention, and more as required during the remainder of the intervention. These data will be reviewed during weekly individual coaching calls. During the 12-month follow-up phase, participants will be encouraged to continue their reduced calorie diet and to monitor daily weights on the Zone of Adherence feedback within the *Companion* App. Once a 10% WL is achieved—be it in the initial 6-month intensive intervention period or the 12-month maintenance phase—the participant will transition to a maintenance goal, reflected by a flat Zone of Adherence.

##### 2.3.2.6 The companion app

The intervention condition will receive an iPad equipped with the *Companion* app toolset, which was refined hand-in-hand with older adults with and without chronic pain (5, 7, 21), and is designed to act as a companion to socially rich in-person and remote WL and daylong PA interventions. The Companion app is study-specific iOS application, with participant data hosted on HIPAA-compliant servers. Notably, participant Fitbit data are associated with anonymized Fitbit accounts (i.e., “Fitbit demo accounts”) to enhance privacy. The first core function of the app is to provide ongoing access to social support, including a private chat with the coach and a group chat feature. Second, the app includes several features designed to support the development of self-efficacy for PA and WL. For instance, participants earn “mastery badges” paired with tailored encouragement in real time as they achieve group-specific goals. As part of the MORPH program, participants learn to view the release of these badges as a cue that they have had success in the program, and they are encouraged to briefly pause and notice the positive sensations that come with this success. We previously demonstrated that this approach to badge delivery can support self-efficacy (50).

**Figure 2:**
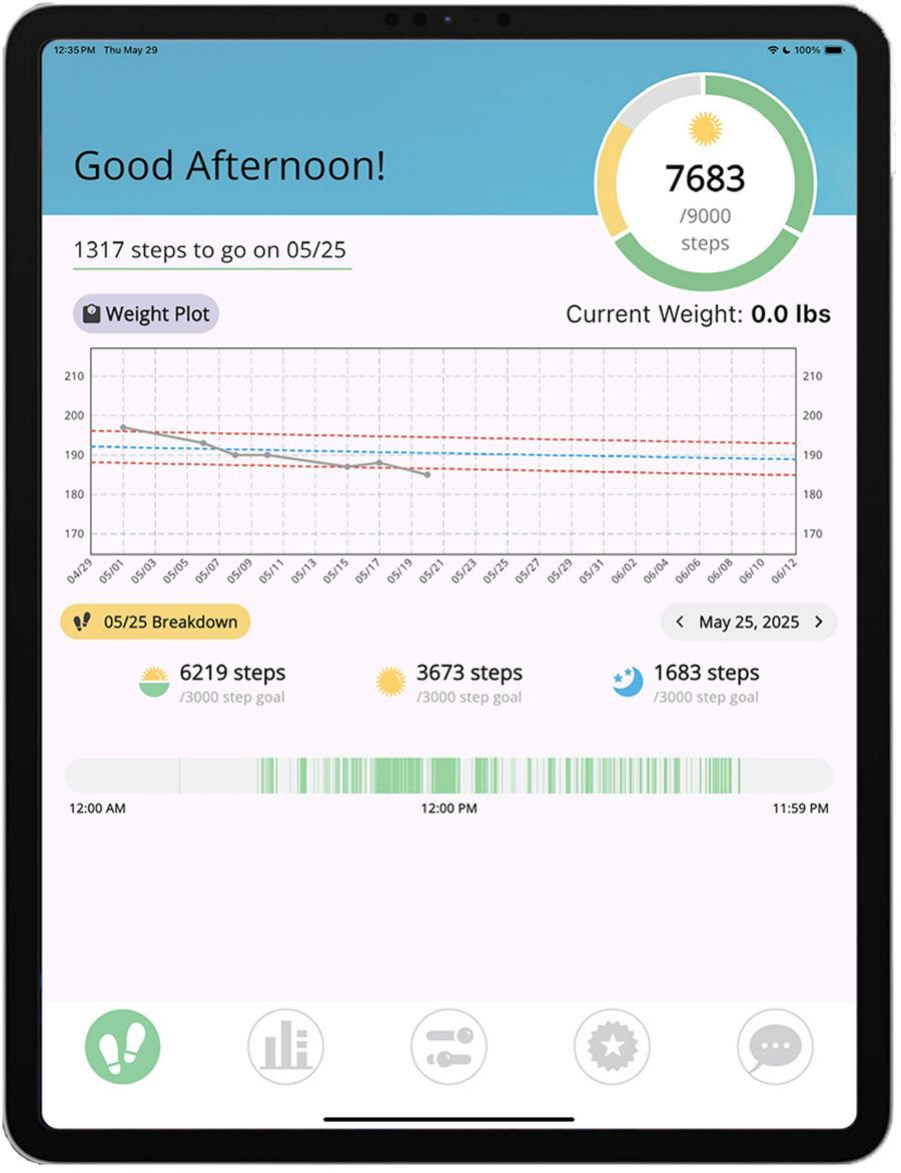
Example companion app.

Third, as an accurate understanding of one's own behavioral patterns is a prerequisite for behavioral self-regulation (51), the app provides unique and objective real-time feedback on patterns of PA and WL over time. The *Companion* app integrates Fitbit data in near real time, with a primary feedback mechanism being a daily “timeline bar” that resides on the bottom of the app's splash screen (see the bottom feedback bar in Figure 2). Here, periods with Fitbit-detected PA are depicted as green stripes. We have found that recommending a “tree rings profile” comprised of frequent stripes of green throughout the full day rather than fewer more sustained periods of green, helps participants to understand the daylong PA goal. The *Companion* app also provides participants with an objective daylong PA goal setting infrastructure via three “periodic step goals.” Here, participants receive an overall daily stepping goal and may earn a portion of their daily “step allowance” before 12:00pm, between 12:01pm and 5:30pm, and after 5:31pm (see pie chart near the top of Figure 2, with each segment representing the day's periods). The portion that can be earned within each period is customized to the daily routines and preferences of the individual while still requiring some movement during each period to achieve the goal (i.e., 100% of the daily goal is not achievable in only one or two periods). Participants are encouraged to view their PA feedback at least once in the morning, midday, and evening to develop an awareness of their own patterns of sitting and moving. Qualitative feedback from the MORPH-II pilot revealed that all but one participant felt this aspect of the app helped them to understand their PA levels. For instance, one participant stated: “It was very helpful to look back and see, ‘Oh my goodness, I sat a really long time.’ … So that was very helpful. I'm going to miss it.” Another noted “The green and blue bar that showed when the activity was more concentrated during the day, and when it wasn't…it was a good visual representation of my activity, and it helped remind me to get up and move more and not sit still for long periods of time.”

The central WL feature is the Zone of Adherence feedback (see the “Weight Plot” in Figure 2) (52), which plots a participant's daily body weights, collected via BodyTrace scale, against their projected WL generated via the NIDDK Body Weight Planner (53), with the aim of achieving 8%–10% WL over 18 months. The plot includes upper and lower fences, allowing for expected variability in body weight while helping to guide participants towards long-term WL. Notably in the recently completed *Healthy Aging and Late-Life Outcomes Pilot* (HALLO-P) trial, we deployed a remotely delivered and group-mediated weight loss and daylong PA intervention similar to the one described herein (though the sample was not required to have chronic pain to participate). Across the 40-week study period, participants accessed the application on 97.8% of days on average; used their wireless scale on 62%–66% of days, though they were asked to taper to once-weekly weighing by the end of the study; and 97% reported satisfaction with both the daylong activity and dietary weight loss aspects of the intervention (29).

#### 2.3.3 Monitoring treatment fidelity and adherence

The nature of the MORPH-III as a technology-driven intervention will facilitate intensive monitoring of adherence and the identification of behavioral lapses early so they may be quickly addressed. It is our belief that high rates of self-monitoring tool use early in the intervention is vital for developing an accurate awareness of one's PA, sedentary behavior, and dietary weight management behaviors. The researcher portal for the *Companion* App provides immediate feedback on when a given participant accessed a tool (e.g., the most recent weight received, app access, or Fitbit synchronization). Individual coaching calls offer an opportunity to review and troubleshoot any synchronization or access issues. Fitbit data will be used to monitor weekly adherence to daylong PA goals, and BodyTrace body weight data will be used to monitor adherence to dietary goals in WL (i.e., whether the participant's weight aligns with their projected WL via the NIDDK body weight planner). Should these data point to possible issues with adherence or goal achievement (e.g., body weight values approach the upper fence of the Zone of Adherence, several sequential days without Fitbit synchronization), the behavioral interventionist may deploy a schedule of increased brief contacts (e.g., one additional weekly brief phone contact for two weeks) to attempt to address these issues early. To bolster coaching fidelity, group sessions and all one-on-one coaching calls will be recorded. Meeting footage will be audited by a study PI at least three times per wave to ensure compliance with the manual of procedures and coaching checklist. More frequent auditing will be utilized early in the study. One PI and the behavioral interventionist will meet after each round of audits to review successes and to raise issues and develop solutions to these issues.

#### 2.3.4 Community engagement

In the spirit of human-centered design and in an effort to ensure the results of this study are broadly generalizable, we will conduct each stage of MORPH-III in collaboration with a CAB and community members (engaged via the CAB) who represent the characteristics of the communities we intend to receive MORPH. The CAB will be formed during study ramp up with the aim of reviewing, informing, and refining study protocol and recruitment efforts. The CAB will consist of approximately 8–10 local lay community members and representatives from community organizations serving older adults with varied socioeconomic backgrounds. Members will be asked to convene approximately quarterly for the duration of the project. As warranted, CAB members will also be asked to participate in small working group sessions focused on study planning and on providing feedback to receipt of intervention content. As data are collected and analyzed, the CAB will play a role in analyzing and interpreting data, as well as developing plans for sharing findings with broader communities. During the final year of the study, a series of blended in-person and digital town hall meetings will be held within key recruitment centers represented by the CAB and external advisors. These meetings will allow for dissemination of research findings back to the communities that supported the project, provide opportunities for community members to give feedback and share thoughts for future research endeavors, and ultimately enhance transparency of the research process and build trust in clinical research.

### 2.4 Measures and associated procedures

Participants will complete key assessments prior to randomization at baseline (BL), following the intensive intervention period (6 months), and following the maintenance period (18 months; 12 months post-intervention). These include the primary outcome of daily steps, the secondary outcome of pain interference, and additional outcomes including body weight, pain intensity and catastrophizing, sedentary behaviors, measures of psychosocial antecedents and consequences of pain and behavior change, quality of life, physical function, and medication usage. The nature, purpose, and risks of all tests will be explained to participants prior to obtaining their written consent. All examiners will be blinded to participant treatment assignment and are trained in the standardized conduct of all tests.

Our study is powered based upon our primary outcome of daily steps. This, alongside other PA and sedentary behavior outcomes, will be assessed via the ActivPAL 4 (PAL Technologies Ltd, Glasgow, UK) accelerometer. The ActivPAL is a small triaxial accelerometer and inclinometer worn on the midline of the non-dominant thigh. We have selected this device as the ActivPAL 4 provides a superior classification of stepping and sedentary behaviors relative to other research monitors such as the ActiGraph (54–56). The ActivPAL 4 also produces excellent wear time as it is sealed in a waterproof sleeve and adhered to the leg using transparent medical dressing. The ActivPAL will be worn for 7 consecutive days prior to randomization, during the final week of the intervention, and during the final week of the maintenance period. Note that a table contrasting roles and measures for the Fitbit and ActivPAL activity monitors can be found in the Supplementary Material.

Secondary outcomes include measures of pain interference, body weight, and physical function. Pain interference (i.e., the extent to which pain interferes with daily life) will be assessed via the 8-item Patient Reported Outcome Measurement Information System (PROMIS) pain interference scale. The 8-item short form scale is recommended by PROMIS and comprises items selected using rankings based on (1) maximal interval information, and (2) computer adaptive testing simulations. This 8-item scale is also responsive to intervention (57). Additionally, we will measure pain intensity (an exploratory measure) via the 3-item PROMIS (58) pain intensity scale. For both PROMIS measures, scores are provided on a t-scale such that 50 represents the national average with a standard deviation of 10. Body weight will be assessed via the BodyTrace scale, which has a stated accuracy of ±100 g and good concordance with clinic assessments of weight (59, 60). Regarding physical function, we will capture leg strength via a remote 30-second chair stand task, and usual gait speed via a 4 m walk task. Each test will be conducted and timed via videoconference while being simultaneously recorded via point-and-shoot video camera to confirm accurate timing, using procedures piloted in the MORPH-II pilot study. We will also use the Pepper Assessment Tool for Disability (PAT-D) (61) to measure subjective function and disability (an exploratory measure).

Finally, we will assess additional exploratory measures to facilitate examination of intervention effects on psychosocial outcomes of interest. We will assess pain catastrophizing using the Pain Catastrophizing Scale (62); a well-validated and widely-used measure of pain catastrophizing (63). We will capture key self-determination and social cognitive theory constructs that are targets of our behavioral intervention. These include self-efficacy for engaging in valued activities despite pain (64), managing eating (65), and for engaging in PA via exercise and through the accumulation of PA across the day (66, 67); outcome expectancies related to PA (68) and nutrition (69); the extent to which the individual experiences satisfaction or frustration in achieving their core needs of social connection, competency, and autonomy (70); the use of self-regulatory strategies for managing eating or PA behavior (69, 71); and perceptions of barriers to healthy eating or engaging in PA (72, 73). We will also assess stress (74) and affect (75), which are key dynamic influences on one's experience of pain. We will collect the 36-item short form health survey (76) as a measure of Health-Related Quality of Life (HRQOL). We will collect the widely-used Pittsburgh Sleep Quality Index (PSQI) as a measure of subjective sleep quality (77, 78). Additionally, at baseline, 6, and 18 months we will query participants about their medication usage in the previous 30 days, which will be inventoried into the following categories: muscle relaxants; opioids; nonsteroidal anti-inflammatory drugs (NSAIDs); anticonvulsants; antidepressants; acetaminophen; aspirin; topical analgesics; and weight loss. Likewise, we will inventory any OA-related procedures (e.g., arthroplasty) done during the intervention. Finally, as in our prior studies (5, 7), we will collect open-response and Likert-style feedback from participants as they exit the 6-month intervention period to further refine the intervention in future applications.

### 2.5 Sample size

The sample size is designed to detect a difference between treatment groups of 1,500 steps per day at 6 months. Using data from those in weight loss and daylong movement condition in the EMPOWER trial (21), the root mean square error (RMSE) was 2,550 steps after baseline adjustment; this is the assumed standard deviation we used for subsequent power calculations. In total, a sample of 124 individuals is required to achieve 90% power. Additionally, the ICC for those who received weight loss and daylong movement in EMPOWER was approximately.04. Assuming sizes for each wave of *n* = 9, the sample size inflation required to account for lack of independence due to waves is 1 + (8 − 1)*0.04 = 1.28. When paired with a conservative estimate of 20% loss-to-follow-up, the required sample size is 200. The 1,500 step per day difference approximates what we have achieved using a progressive goal setting infrastructure in only 3 months in our most updated protocol (MORPH-II); recognizing that goals would continue to increase across the 6-month focused intervention period. Additionally, across both MORPH studies, baseline average daily steps were 4,812; an increase of 1,500 steps is sufficient to achieve a 6,000 step/day target recommended for sustaining function in individuals with knee OA (14). Finally, this target aligns with minimally clinically meaningful improvements in other pain populations [1,211 steps for enhancing HRQOL in adults with peripheral artery disease (79), and 1,000 steps per day for enhancing HRQOL in adults with fibromyalgia] (80).

### 2.6 Analyses

To investigate the impact of the intervention on daily steps relative to control at 6 months, we will conduct a mixed-model repeated measures analysis of covariance with an unstructured covariance matrix to account for the fact that multiple measurements within a participant over time are not independent. We focus on the contrast at 6 months but will include all time points (i.e., 6 and 18 months), to improve power, particularly if values are missing at month 6. Models will use restricted maximum likelihood (REML) with unstructured covariance. REML handles missing data by utilizing all available data under the assumption of missing at random. Recruitment wave will be included as a random effect to adjust for the grouped intervention, and both sex and baseline value of the dependent variable of interest will be included as fixed effects. Inclusion of baseline is consistent with regulatory guidance (81), will increase efficiency (82, 83), and is preferred in randomized controlled trials (84). All tests of hypotheses and reported *p* values will be two-sided using the intention to treat principle. Subgroups of interest include sex, race, and ethnicity. Formal tests of subgroup differences will be made using tests of interactions between the subgroup and treatment to examine whether these variables moderate the treatment effect. A similar analytic approach will be used for the secondary and exploratory outcomes. In a final exploratory model, we will examine the possibility that self-efficacy for engaging in PA across the day mediates the relationship between treatment and change in steps (if present) by adding the change in self-efficacy to the primary model.

## 3 Discussion

Sustaining functional independence and HRQOL are key healthcare targets for older adults (85, 86). Unfortunately, these outcomes are powerfully affected by the experience of chronic pain; an age-related condition that predisposes older adults to weight gain, inactivity, and social isolation that in turn exacerbate pain-related symptoms. Opioid use—a common pain management strategy—increases the risk of addiction and accompanying complications. A promising behavioral medicine approach for those living with pain is the combination of the accumulation of PA throughout the day and weight loss. These behaviors are associated with reduced inflammation and improved pain symptoms (87–89).

The overarching goal of *MORPH-III* is to test the efficacy of an intervention designed to enhance PA via steps and to improve pain interference, body weight, and physical function among older adults with chronic knee or hip OA pain. It builds upon lessons learned in two iterative refinement trials (5, 7) and represents a key next step in the development of PA promotion interventions that encourage individuals to think about PA as a behavior that occurs throughout the day. Specifically, it addresses key gaps raised in a meta-analysis of interventions targeting brief bouts of activity by Jones and colleagues (27): trials to date are limited in number and comprise small quasi-experimental studies, small randomized controlled trials, or qualitative studies, and none targeted pain outcomes. If successful, the results of MORPH-III may affect the way in which both activity promotion professionals and those working with individuals with chronic pain approach PA behavior change. Specifically, it is our hope that successful results of this trial will encourage further research and public interest in adopting diverse active lifestyles and a reduced emphasis on promoting a select few types of structured exercise.

### 3.1 Strengths

There are several key strengths to the proposed trial. Our approach to enhancing PA is novel and may be a more approachable and sustainable method for building an active lifestyle, particularly among those with chronic pain. Second, it is sufficiently powered for our primary outcome and boasts both a relatively long (6 months) intervention and follow-up period (12 months for a total of 18 months), which is important for ensuring PA changes are not due to novelty and for examining durability of effects on health behaviors, pain, and functioning. Third, as a fully remotely delivered intervention that provides cellular enabled technology if needed, individuals can participate regardless of geographic location or access to technologies. Finally, the protocol is built upon a series of iterative user-centered design studies and is supported by a CAB, which improves the likelihood the intervention is seen as feasible and valuable by those who participate.

### 3.2 Limitations

As a conventional two-arm randomized controlled trial, there are several notable limitations that may be addressed by additional research. For instance, we will be unable to disentangle the effects of the group and coach contact or changes in body mass on physical activity behaviors, nor how these factors affect outcomes like pain interference or catastrophizing. Additionally, as with many behavioral trials, there are likely many older adults with chronic pain who do not feel ready to change their behavior and as such are not reached by our program. Additional work encouraging readiness among these individuals is warranted. Finally, the requirement for a trained group leader and access to objective monitoring technologies may be a barrier to future scale, and as such additional implementation work is required to establish pathways toward future deployment (e.g., via insurance coverage for wearable monitoring).

### 3.3 Conclusion

Osteoarthritis is a leading cause of debilitating pain among older adults (1, 2), especially those living with obesity (15). Reducing adiposity and adopting a physically active lifestyle marked by sufficient levels of activity done often throughout the day are associated with better pain management. Unfortunately, few older adults engage in sufficient activity (90) and nearly 40% live with obesity (91), and pain itself represents an important barrier to activity and dietary behavior change. Should the MORPH-III trial demonstrate efficacy of a remotely delivered daylong movement and healthy eating intervention, it will set the stage for real-world efficacy and effectiveness testing with the long-range goal of establishing a scalable and sustainable lifestyle approach to pain management for those living with chronic pain.

## Data Availability

The original contributions presented in the study are included in the article/supplementary material, further inquiries can be directed to the corresponding author/s.
