# Supplementary material for: A mobile intervention to reduce pain and improve health-III: protocol for a remotely delivered randomized controlled trial of physical activity for pain management in older adults with obesity and knee or hip osteoarthritis
Source: Front Digit Health. 2026 May 21;8:1739501. doi: 10.3389/fdgth.2026.1739501 (PMC13234624; doi:10.3389/fdgth.2026.1739501)
Supplement: Supplementary file 1 [file Supplementaryfile1.docx]

Supplementary Material

# Full Inclusion and Exclusion Criteria

## Inclusion Criteria

Participants must meet the following inclusion criteria to participate in the study:

- Aged 65+ years
- Body mass index of 30-45 kg/m2 or >45 kg/m2 with physician’s consent
- No loss or gain of more than 5% body mass in previous 6 months
- Presence of knee or hip osteoarthritis as ascertained via the Roux questionnaire
- Independently living
- Low active (i.e., not participating in regular resistance training and/or > 20 mins/day of aerobic exercise on more than 2 days/week in past 3 months as ascertained via a modified CHAMPS questionnaire)
- Have no contraindication for safe and optimal participation in exercise based on EASY screening
- Not currently using a weight loss medication
- Approved for participation by medical director
- Willing to provide informed consent and agree to all study procedures and assessments.

## Exclusion Criteria

Participants meeting any of the following exclusion criteria will not be admitted into the study:

- Reside in skilled nursing facility, rehab or assisted living environment
- History of pharmacologic treatment for a psychiatric disorder other than depression/anxiety within past year
- Current untreated and/or unstable clinical depression or anxiety (Patient Health Questionnaire (PHQ-9) >15)
- Hospitalization for psychiatric event within past year prior to screening
- History of mild cognitive impairment or dementia
- Cognitive impairment (<32) on Modified Telephone Interview for Cognitive Status survey (TICS-M)
- Hearing or visual impairment that would preclude use of the videoconferencing software
- Severe arthritis or other musculoskeletal disorder that is a contraindication for safe walking
- Presently undergoing treatment for orthopedic fracture
- Currently using a weight loss medication
- Contraindication based on EASY screening without physician approval.
- Joint replacement or other orthopedic surgery in past 6 months
- Joint replacement or other orthopedic surgery planned in next 18 months
- Have a diagnosis of uncontrolled hypertension; current or recent past (within 1 year) severe symptomatic heart disease, uncontrolled angina, stroke, chronic respiratory disease other than asthma or COPD, any disease requiring oxygen use, neurological or hematological disease; cancer requiring current treatment, except non-melanoma skin cancers; kidney failure requiring dialysis; have a Katz ADL disability; or engage in heavy alcohol use >14 drinks/week.
- Current participation in other research study with a prospective intervention
- Unable/unwilling to commit to study protocol, including random assignment and use of technology tools
- Unable/unwilling to attend three virtual testing appointments

# 2. Fitbit and ActivPAL Activity Data Collection

| Device | Purpose | Key Metrics |
| --- | --- | --- |
| Fitbit Charge 5 | To facilitate visualization of within-day stepping behaviors | Minute-levels steps |
| ActivPAL 4 | Assessment of physical activity behaviors | Steps (primary outcome)  Sedentary time  Postural shifts  Time spent stepping at 100+ steps per minute  Sleep duration |
